# Performance of sparse-view CT reconstruction with multi-directional gradient operators

**DOI:** 10.1371/journal.pone.0209674

**Published:** 2019-01-07

**Authors:** Chia-Jui Hsieh, Shih-Chun Jin, Jyh-Cheng Chen, Chih-Wei Kuo, Ruei-Teng Wang, Woei-Chyn Chu

**Affiliations:** 1 Department of Biomedical Engineering, National Yang-Ming University, Taipei, Taiwan, R.O.C; 2 Department of Biomedical Imaging & Radiological Sciences, National Yang-Ming University, Taipei, Taiwan, R.O.C; 3 Materials & Electro-Optics Research Division, National Chung-Shan Institute of Science & Technology, Taoyuan, Taiwan, R.O.C; 4 Department of Electro-Optical Engineering, National United University, Miaoli County, Taiwan, R.O.C; Beijing University of Technology, CHINA

## Abstract

To further reduce the noise and artifacts in the reconstructed image of sparse-view CT, we have modified the traditional total variation (TV) methods, which only calculate the gradient variations in *x* and *y* directions, and have proposed 8- and 26-directional (the multi-directional) gradient operators for TV calculation to improve the quality of reconstructed images. Different from traditional TV methods, the proposed 8- and 26-directional gradient operators additionally consider the diagonal directions in TV calculation. The proposed method preserves more information from original tomographic data in the step of gradient transform to obtain better reconstruction image qualities. Our algorithms were tested using two-dimensional Shepp–Logan phantom and three-dimensional clinical CT images. Results were evaluated using the root-mean-square error (RMSE), peak signal-to-noise ratio (PSNR), and universal quality index (UQI). All the experiment results show that the sparse-view CT images reconstructed using the proposed 8- and 26-directional gradient operators are superior to those reconstructed by traditional TV methods. Qualitative and quantitative analyses indicate that the more number of directions that the gradient operator has, the better images can be reconstructed. The 8- and 26-directional gradient operators we proposed have better capability to reduce noise and artifacts than traditional TV methods, and they are applicable to be applied to and combined with existing CT reconstruction algorithms derived from CS theory to produce better image quality in sparse-view reconstruction.

## Introduction

Since the mathematical model of image reconstruction was proposed by Radon in 1917 and the X-ray computed tomography (CT) scanner was invented by Hounsfield in 1972 [1–3], CT technology has been widely employed in various clinical institutions because it is noninvasive and allows observation of the internal structure of the human [4–7]. Studies related to X-ray CT have flourished in the past three decades [8–15].

Because of the arising in health awareness, an increasing number of people have become concerned about the radiation dose in using X-ray CT [16–19]. Studies have reported that an excessive X-ray dose increases the risk of tissue diseases and cancers [20–23]. How to reduce X-ray dose in CT while maintaining image quality has thus been a highly active research topic in the past decades [24–27].

Two methods are typically used to reduce X-ray dose in CT. The first is to reduce the X-ray exposure time and the current or voltage of the X-ray tube at each sampling step; the second is to reduce the sampling density during the CT scan, i.e., reducing the amount of projection data. Both methods would eventually introduce higher noise that renders poor image quality.

Since the theory of compressed sensing (CS) was proposed by Candès, Romberg, Tao, and Donoho in 2006 [28], studies have sought to reconstruct high-quality medical images when insufficient data are available [29–45]. CS is a theory for reconstructing original signals (images) when the number of samples (amount of data) is insufficient. Using CS to reconstruct CT images from insufficient data is an optimization problem. For solving this kind of problem, minimizing a L1 norm from the sparse representation of the original image is a typical way.

In 2008, Sidky and Pan proposed a reconstruction method which used the total variation (TV) of an image to represent the sparse representation and minimize the L1-norm of TV to solve the optimization problem in the iteration process[29]. They added a TV term in the process of reconstruction to constraint the image convergence, and the steepest descent method was used to solve the optimization problem. After a number of iterations, a better quality image can be reconstructed. The TV term mentioned in this method is to calculate the gradient in *x* and *y* directions by gradient operator. In 2009, Sidky and Pan proved the feasibility of applying the TV method to CT reconstruction in few-views and limited-angle situation[30]. Based on this, various improved TV have been proposed to increase the quality of reconstructed image. For example, the PICCS (prior image constrained compressed sensing) proposed by Chen GH and Leng S, not only used TV to constraint the convergence of iteration but also added the information from prior image in the reconstruction process, so that the reconstructed images after convergence were closer to the original image[31]. Furthermore, Yu H and Wang G proposed an algorithm for solving interior tomography by TV method in 2009[32]. This research used minimization of the image TV to reconstruct the region of interest (ROI) without reconstructing the entire image, which greatly reduced the computing resources and reconstruction time. In above studies, although the TV method can effectively reduce noise and artifacts caused by the spare-view situation and reconstruct the image with high quality, the oversmoothing problem at edge parts in the image is inevitable. In order to solve this problem, Tian Z and Jiang SB proposed an improved TV method which could preserve the edge parts in the image, referred as EPTV (edge-preserving total variation)[33]. In order to allow the effect of TV smoothing to be applied only on the non-edge parts, EPTV gave the edge and non-edge parts a different penalty weight during the reconstruction process. Because a smaller penalty weight was given to the edge parts, the resolution of edge could be preserved. For mitigating the edge blurring effect caused by the TV method, Liu Y et al. proposed a AwTV (adaptive-weighted TV) to overcome the problem[34]. By considering the anisotropic edge property among neighboring image voxels, this method added exponential weights to the traditional TV term, and automatically adjusted the weight ratio according to the gradient of the image to preserve the edge details in the image.

In the course of developing the TV methods for various low-dose situations and applications, we found that all of the above-mentioned TV methods only calculate the gradient variations in the *x* and *y* directions. Whether the gradient information of the original tomographic data could be better preserved by considering additional gradient variations besides only in *x* and *y* directions was a question worthy of further study. Deng L et al. proposed a diagonal TV calculation method in 2015 to improve the quality of CT image reconstruction in the case of sparse-view[35]. Instead of calculating the *x* and *y* direction, they calculated only the gradient variations of four diagonal directions to transform the image into sparse representation and solved the optimization problem. They claimed that the diagonal TV method actually reconstructs better images than traditional TV method which only considers the directions in *x* and *y*. Encouraged by this research, we began to think about the correlation between using more number of directions in TV calculation and improving the quality of image. From the conventional calculation of TV which only *x* and *y* directions were considered, to the four directions in TV calculation which included both positive and negative directions of *x* and *y*, and then further to the 8-directional TV method in which the gradient variations in the additional four diagonal directions were taken into consideration. As the number of the directions in TV calculation increases, the sparse image (sparse representation) converted by the gradient operator could preserve more information from the original tomographic data, so that the subsequent minimization of the sparse representation of its L1 norm to solve the optimization problem could obtain a better solution. It is the issue that this study wants to explore in depth. If using more number of directions in TV calculation can effectively improve the quality of reconstructed images, all of the above algorithms using traditional 2- or 4-directional TV methods have the opportunity to improve their algorithms by adding another four diagonal directions in TV calculation (total of 8-directional TV calculation). Furthermore, the proposed 8-directional TV method is applicable to be applied to all of the above reconstruction algorithms and have better results.

This paper proposes a gradient operator that considers the calculation of TV in the diagonal directions for the gradient transform of two-dimensional (2D) images. In contrast to the traditional gradient operators, which only calculate TV in two or four directions (both the positive and negative directions of the *x*- and *y*-axes), this paper proposes a multi-directional gradient operator for two-dimensional (2D) images that additionally calculates TV in four diagonal directions. The 8-directional gradient operator ensures a better gradient transform. It preserves more information than traditional gradient operators, and high-quality images can thus be subsequently reconstructed using a CS-based image reconstruction algorithm. Moreover, this paper also develops a gradient operator that involves 26 directions for three-dimensional (3D) images to increase the quality of 3D sparse-view CT images.

In the remainder of this paper, Section II introduces the calculation principles and methods for the 8- and 26-directional gradient operators and delineates the procedures of the sparse-view image reconstruction algorithms based on the calculation of TV in multiple directions. Section III presents the results of simulated and actual images reconstructed using the proposed multi-directional gradient operators, and the results are compared with those obtained using the traditional 2- and 4-directional gradient operators as well as 3- and 6- directional gradient operators. Finally, Sections IV and V present the discussion and conclusions.

## Materials and methods

### CS-based CT reconstruction theory

Theoretically, CT imaging can be expressed in the following mathematical equation:

$$P\vec{f}=\vec{x}$$ (1)

where $\vec{f}$ is the original image to be reconstructed; *P* is the Radon operator of forward projection during the CT scan; and $\vec{x}$ denotes the projection data obtained after the CT scan. Traditional CT reconstruction algorithms, such as filtered back projection (FBP), tend to generate severe noise and artifacts in images and fail to reconstruct high-quality images if low-density sampling were used. The improvement in image quality that can be obtained using subsequently developed iterative reconstruction algorithms such as the algebraic reconstruction technique (ART), the simultaneous iterative reconstruction technique (SIRT), and the simultaneous ART (SART). Nevertheless, improvements in image quality are limited if low-density sampling was employed.

The theory of CS enables high-quality CT images to be reconstructed when sampling-density was sparse [28]. According to CS theory, a signal can be effectively reconstructed when its sampling frequency is considerably lower than that required by Nyquist–Shannon sampling theory providing that the signal is a sparse representation in a specific domain. Sparse representation is defined as more signal values equal to zero than values not equal to zero. The process of transforming a signal into the sparse domain is called the sparse transform, and gradient transform is a type of sparse transform that is commonly used in CT image reconstruction.

The theory of CS can be expressed as follows:

$$\min\;\|\vec{y}{\|}_{1}\;s.t.\;\vec{x}=P\vec{f}=P\Phi \vec{y}$$ (2)

where Φ denotes sparse transform, which refers to gradient transform in this study. In addition, $\vec{y}$ is the sparse representation of the original image $\vec{f}$ in the sparse domain and *P* is the Radon operator in the forward projection. The CS-based reconstruction is aimed at finding $\vec{y}$ in sparse domain by solving Eq (2).

### Reconstruction algorithm for 2- or 4-directional gradient operator

Inspired by the CS theory, Sidky and Pan proposed a CT reconstruction algorithm based on TV calculation [29] in which the gradient operator was defined as follows:

$$\mu_{m,n}=\sqrt{{\left(f_{m,n}-f_{m-1,n}\right)}^{2}+{\left(f_{m,n}-f_{m,n-1}\right)}^{2}}$$ (3)

$$f_{TV}=\sum_{m,n}\mu_{m,n}$$ (4)

where *f*_*TV*_ is the TV of image $\vec{f}$ and also the sparse representation of the image after sparse transform; *f*_*m*,*n*_ is a pixel in $\vec{f}$; and *m* and *n* represent the pixel row and column of the image, respectively.

In this algorithm, gradient transform only calculates the TV in two directions. To improve the quality of the constructed images, Yu and Wang proposed a CS-based CT reconstruction algorithm that uses a 4-directional gradient operator for gradient transform [32]. According to their method, an approximation of the gradient operator in image gradient transform can be expressed as follows:

$$\mu_{m,n}≅\sqrt{\frac{{\left(f_{m+1,n}-f_{m,n}\right)}^{2}+{\left(f_{m,n}-f_{m-1,n}\right)}^{2}+{\left(f_{m,n+1}-f_{m,n}\right)}^{2}+{\left(f_{m,n}-f_{m,n-1}\right)}^{2}}{2\Delta^{2}}+\varepsilon^{2}}$$ (5)

where Δ indicates the sampling interval and ε is the constant added to prevent the denominator from being zero in the calculation of steepest descent direction. The TV is defined as follows:

$$f_{TV}=\sum_{m,n}\mu_{m,n}$$ (6)

Therefore, the steepest descent direction can be obtained using

$$d_{m,n}=\frac{\partial f_{TV}}{\partial f_{m,n}}=\frac{4f_{m,n}-f_{m+1,n}-f_{m-1,n}-f_{m,n+1}-f_{m,n-1}}{\mu_{m,n}}+\frac{f_{m,n}-f_{m+1,n}}{\mu_{m+1,n}}+\frac{f_{m,n}-f_{m-1,n}}{\mu_{m-1,n}}+\frac{f_{m,n}-f_{m,n+1}}{\mu_{m,n+1}}+\frac{f_{m,n}-f_{m,n-1}}{\mu_{m,n-1}}$$ (7)

Steepest descent direction can be regarded as the correction term for each iteration in the CS-based reconstruction algorithm. The entire pseudo code of the reconstruction algorithm is presented in Yu and Wang's study [32].

### CS-based reconstruction algorithm for 8- or 26-dimensional gradient operator

Inspired by the aforementioned two studies, we reason that using more directions for TV calculation can further improve the quality of the CT images. Taking a 2D image as an example, each pixel has eight pixels surrounding it; therefore, one can use up to eight directional gradients to calculate the TV (named as an 8-directional gradient operator) to sparse transform the 2D image. Because the proposed method considers the gradients between all surrounding pixels and the pixel of interest, the calculated steepest descent direction is more accurate, which is favorable because it prevents the generation of noise and artifacts in each iteration.

The 8-directional gradient operator is expressed as follows:

$$\mu_{(8)m,n}≅\sqrt{\begin{array}{c} \frac{{\left(f_{m+1,n}-f_{m,n}\right)}^{2}+{\left(f_{m,n}-f_{m-1,n}\right)}^{2}+{\left(f_{m,n+1}-f_{m,n}\right)}^{2}}{2\Delta^{2}}+\\ \frac{{\left(f_{m,n}-f_{m,n-1}\right)}^{2}+{\left(f_{m+1,n-1}-f_{m,n}\right)}^{2}+{\left(f_{m+1,n+1}-f_{m,n}\right)}^{2}}{2\Delta^{2}}+\\ \frac{{\left(f_{m,n}-f_{m-1,n-1}\right)}^{2}+{\left(f_{m,n}-f_{m-1,n+1}\right)}^{2}}{2\Delta^{2}}+\varepsilon^{2} \end{array}}$$ (8)

$$f_{(8)TV}=\sum_{m,n}\mu_{(8)m,n}$$ (9)

where *f*_(8)*TV*_ is the TV of the image calculated using the 8-directional gradient operator. The steepest descent direction can be further obtained using

$$d_{(8)m,n}=\frac{\partial f_{(8)TV}}{\partial f_{m,n}}=\frac{8f_{m,n}-f_{m+1,n}-f_{m-1,n}-f_{m,n+1}-f_{m,n-1}-f_{m+1,n-1}-f_{m+1,n+1}-f_{m-1,n-1}-f_{m-1,n+1}}{\mu_{(8)m,n}}+\frac{f_{m,n}-f_{m+1,n}}{\mu_{(8)m+1,n}}+\frac{f_{m,n}-f_{m-1,n}}{\mu_{(8)m-1,n}}+\frac{f_{m,n}-f_{m,n+1}}{\mu_{(8)m,n+1}}+\frac{f_{m,n}-f_{m,n-1}}{\mu_{(8)m,n-1}}+\frac{f_{m,n}-f_{m+1,n-1}}{\mu_{(8)m+1,n-1}}+\frac{f_{m,n}-f_{m+1,n+1}}{\mu_{(8)m+1,n+1}}+\frac{f_{m,n}-f_{m-1,n-1}}{\mu_{(8)m-1,n-1}}+\frac{f_{m,n}-f_{m-1,n+1}}{\mu_{(8)m-1,n+1}}$$ (10)

**Fig 1:**
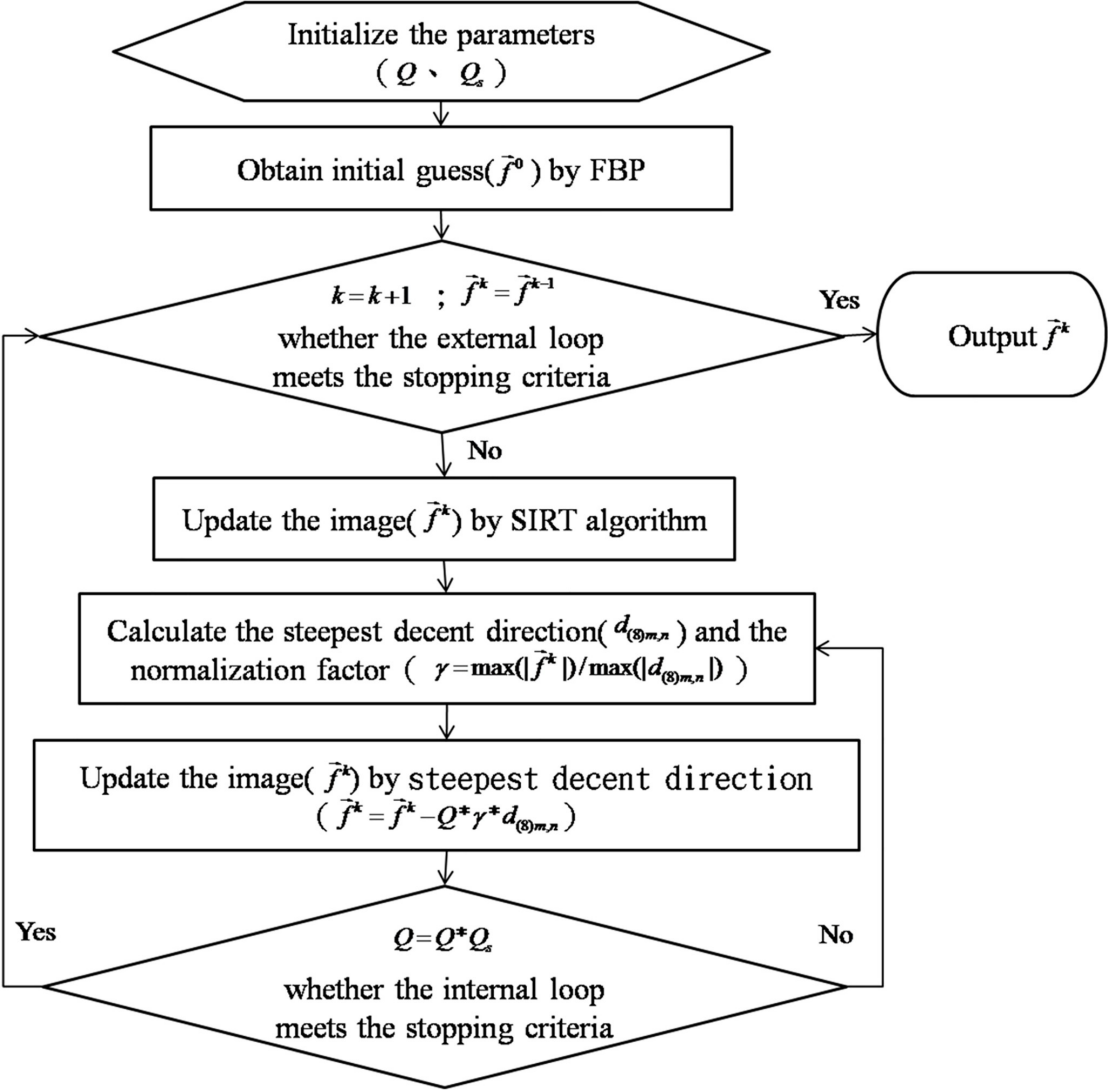
Procedure of compressed-sensing-based reconstruction algorithm using the 8-directional gradient operator. FBP: filtered back projection. SIRT: simultaneous iterative reconstruction technique.

The obtained *d*_(8)*m*,*n*_ is used as a correction in the CS-based 2D image reconstruction algorithm, the procedure of which is displayed in Fig 1.

In Fig 1, parameter *Q* is the level (or ratio) of steepest descent and *Q*_*s*_ is the parameter representing the ratio of the descent of *Q* after each internal loop. These two parameters are given a fixed value before the image reconstruction begins. This algorithm reconstructs projection data using the traditional FBP at first. However, an image reconstructed by FBP contains a large amount of noise and artifacts because of the low-density sampling. Despite its low quality, the reconstructed image does rudimentarily represent the original image and is thus used as the initial guess required for subsequent SIRT to reduce the number of iterations. At this step, whether the external loop meets the stopping criteria should be determined. The fixed number of iterations set initially is used as the stopping criterion. When the preset number of iterations is achieved in the external loop, ${\vec{f}}^{k}$ is output as the reconstruction result. If the stopping criterion for the external loop is not satisfied, SIRT is performed to correct ${\vec{f}}^{k}$ once. Subsequently, the algorithm proceeds to the internal loop, in which the steepest descent direction method is used to minimize L1 norm. In the internal loop, an iteration is performed as follows. Eqs (8)–(10) are used to calculate the steepest descent direction *d*_(8)*m*,*n*_, and the normalization factor γ is then calculated according to the ratio of the maximum absolute values of ${\vec{f}}^{k}$ and *d*_(8)*m*,*n*_. The normalized steepest descent direction *γ* * *d*_(8)*m*,*n*_ is multiplied by the level of steepest descent *Q* to produce the correction item which is used to correct ${\vec{f}}^{k}$. This completes one iteration. The stopping criterion for the internal loop is also the preset number of iterations. At the end of each internal loop, *Q* is multiplied by *Q*_*s*_ to be reduced by a certain ratio. Images reconstructed with this algorithm are presented in the next section.

**Fig 2:**
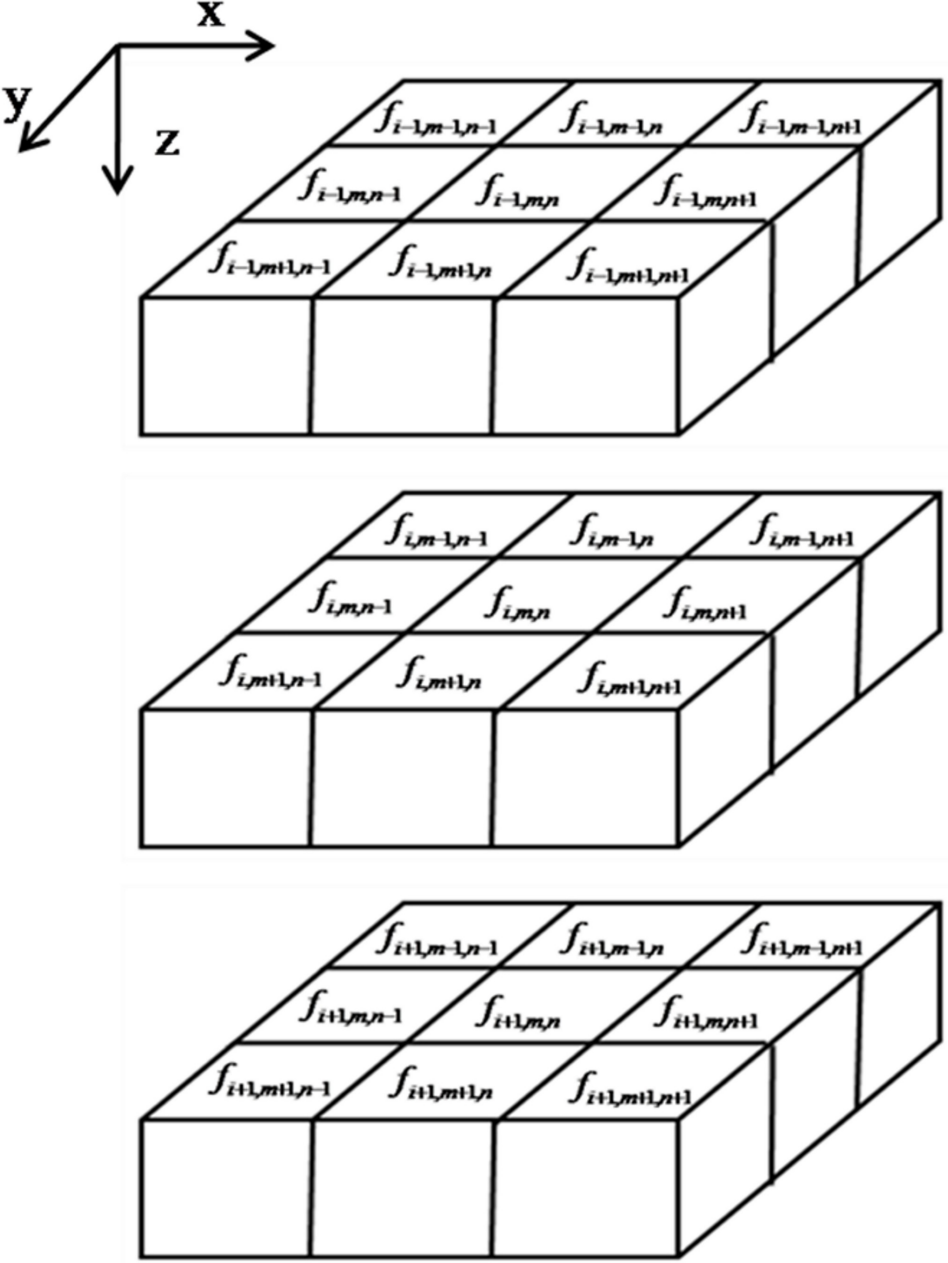
Relative positions of voxels in a 3D image. *m* and *n* represent the voxel row and column of the image. *i* is the level in the *z* direction. The voxel *f*_*i*,*m*,*n*_ has 26 neighboring voxels around it.

For the reconstruction of 3D images, the number of directions used in the gradient operator can be increased in a way similar to those in the 2D images, i.e., 26, to improve reconstructed image quality. This is illustrated in Fig 2, which shows the relative positions of voxels in a 3D image from the *i* − 1 level to the *i* + 1 level in the *z* direction. The voxel *f*_*i*,*m*,*n*_ has 26 neighboring voxels, therefore, the gradient operator involves 26 directions and the steepest descent direction can be calculated as follows:

$$\mu_{(26)i,m,n}≅\sqrt{\begin{array}{c} \frac{{\left(f_{i,m,n}-f_{i-1,m-1,n-1}\right)}^{2}+{\left(f_{i,m,n}-f_{i-1,m-1,n}\right)}^{2}+{\left(f_{i,m,n}-f_{i-1,m-1,n+1}\right)}^{2}}{2\Delta^{2}}+\\ \frac{{\left(f_{i,m,n}-f_{i-1,m,n-1}\right)}^{2}+{\left(f_{i,m,n}-f_{i-1,m,n}\right)}^{2}+{\left(f_{i,m,n}-f_{i-1,m,n+1}\right)}^{2}}{2\Delta^{2}}+\\ \frac{{\left(f_{i,m,n}-f_{i-1,m+1,n-1}\right)}^{2}+{\left(f_{i,m,n}-f_{i-1,m+1,n}\right)}^{2}+{\left(f_{i,m,n}-f_{i-1,m+1,n+1}\right)}^{2}}{2\Delta^{2}}+\\ \frac{{\left(f_{i,m+1,n}-f_{i,m,n}\right)}^{2}+{\left(f_{i,m,n}-f_{i,m-1,n}\right)}^{2}+{\left(f_{i,m,n+1}-f_{i,m,n}\right)}^{2}}{2\Delta^{2}}+\\ \frac{{\left(f_{i,m,n}-f_{i,m,n-1}\right)}^{2}+{\left(f_{i,m+1,n-1}-f_{i,m,n}\right)}^{2}+{\left(f_{i,m+1,n+1}-f_{i,m,n}\right)}^{2}}{2\Delta^{2}}+\\ \frac{{\left(f_{i,m,n}-f_{i,m-1,n-1}\right)}^{2}+{\left(f_{i,m,n}-f_{i,m-1,n+1}\right)}^{2}+{\left(f_{i+1,m-1,n-1}-f_{i,m,n}\right)}^{2}}{2\Delta^{2}}+\\ \frac{{\left(f_{i+1,m-1,n}-f_{i,m,n}\right)}^{2}+{\left(f_{i+1,m-1,n+1}-f_{i,m,n}\right)}^{2}+{\left(f_{i+1,m,n-1}-f_{i,m,n}\right)}^{2}}{2\Delta^{2}}+\\ \frac{{\left(f_{i+1,m,n}-f_{i,m,n}\right)}^{2}+{\left(f_{i+1,m,n+1}-f_{i,m,n}\right)}^{2}+{\left(f_{i+1,m+1,n-1}-f_{i,m,n}\right)}^{2}}{2\Delta^{2}}+\\ \frac{{\left(f_{i+1,m+1,n}-f_{i,m,n}\right)}^{2}+{\left(f_{i+1,m+1,n+1}-f_{i,m,n}\right)}^{2}}{2\Delta^{2}}+\varepsilon^{2} \end{array}}$$ (11)

$$d_{(26)i,m,n}=\frac{\partial f_{(26)TV}}{\partial f_{i,m,n}}=\frac{26f_{i,m,n}-f_{i-1,m-1,n-1}-f_{i-1,m-1,n}-f_{i-1,m-1,n+1}-f_{i-1,m,n-1}-f_{i-1,m,n}-f_{i-1,m,n+1}}{\mu_{(26)i,m,n}}-\frac{f_{i-1,m+1,n-1}-f_{i-1,m+1,n}-f_{i-1,m+1,n+1}-f_{i,m+1,n}-f_{i,m-1,n}-f_{i,m,n+1}-f_{i,m,n-1}-f_{i,m+1,n-1}}{\mu_{(26)i,m,n}}-\frac{f_{i,m+1,n+1}-f_{i,m-1,n-1}-f_{i,m-1,n+1}-f_{i+1,m-1,n-1}-f_{i+1,m-1,n}-f_{i+1,m-1,n+1}-f_{i+1,m,n-1}}{\mu_{(26)i,m,n}}-\frac{f_{i+1,m,n}-f_{i+1,m,n+1}-f_{i+1,m+1,n-1}-f_{i+1,m+1,n}-f_{i+1,m+1,n+1}}{\mu_{(26)i,m,n}}+\frac{f_{i,m,n}-f_{i-1,m-1,n-1}}{\mu_{(26)i-1,m-1,n-1}}+\frac{f_{i,m,n}-f_{i-1,m-1,n}}{\mu_{(26)i-1,m-1,n}}+\frac{f_{i,m,n}-f_{i-1,m-1,n+1}}{\mu_{(26)i-1,m-1,n+1}}+\frac{f_{i,m,n}-f_{i-1,m,n-1}}{\mu_{(26)i-1,m,n-1}}+\frac{f_{i,m,n}-f_{i-1,m,n}}{\mu_{(26)i-1,m,n}}+\frac{f_{i,m,n}-f_{i-1,m,n+1}}{\mu_{(26)i-1,m,n+1}}+\frac{f_{i,m,n}-f_{i-1,m+1,n-1}}{\mu_{(26)i-1,m+1,n-1}}+\frac{f_{i,m,n}-f_{i-1,m+1,n}}{\mu_{(26)i-1,m+1,n}}+\frac{f_{i,m,n}-f_{i-1,m+1,n+1}}{\mu_{(26)i-1,m+1,n+1}}+\frac{f_{i,m,n}-f_{i,m+1,n}}{\mu_{(26)i,m+1,n}}+\frac{f_{i,m,n}-f_{i,m-1,n}}{\mu_{(26)i,m-1,n}}+\frac{f_{i,m,n}-f_{i,m,n+1}}{\mu_{(26)i,m,n+1}}+\frac{f_{i,m,n}-f_{i,m,n-1}}{\mu_{(26)i,m,n-1}}+\frac{f_{i,m,n}-f_{i,m+1,n-1}}{\mu_{(26)i,m+1,n-1}}+\frac{f_{i,m,n}-f_{i,m+1,n+1}}{\mu_{(26)i,m+1,n+1}}+\frac{f_{i,m,n}-f_{i,m-1,n-1}}{\mu_{(26)i,m-1,n-1}}+\frac{f_{i,m,n}-f_{i,m-1,n+1}}{\mu_{(26)i,m-1,n+1}}+\frac{f_{i,m,n}-f_{i+1,m-1,n-1}}{\mu_{(26)i+1,m-1,n-1}}+\frac{f_{i,m,n}-f_{i+1,m-1,n}}{\mu_{(26)i+1,m-1,n}}+\frac{f_{i,m,n}-f_{i+1,m-1,n+1}}{\mu_{(26)i+1,m-1,n+1}}+\frac{f_{i,m,n}-f_{i+1,m,n-1}}{\mu_{(26)i+1,m,n-1}}+\frac{f_{i,m,n}-f_{i+1,m,n}}{\mu_{(26)i+1,m,n}}+\frac{f_{i,m,n}-f_{i+1,m,n+1}}{\mu_{(26)i+1,m,n+1}}+\frac{f_{i,m,n}-f_{i+1,m+1,n-1}}{\mu_{(26)i+1,m+1,n-1}}+\frac{f_{i,m,n}-f_{i+1,m+1,n}}{\mu_{(26)i+1,m+1,n}}+\frac{f_{i,m,n}-f_{i+1,m+1,n+1}}{\mu_{(26)i+1,m+1,n+1}}$$ (12)

The procedure for 3D image reconstruction is similar to that presented in Fig 1; however, the initial guess is obtained using the Feldkamp-Davis-Kress (FDK) algorithm, which is specifically used for the 3D image reconstruction, and *d*_(8)*m*,*n*_ is replaced with *d*_(26)*i*,*m*,*n*_ to minimize the L1 norm of a 3D image. The results obtained for 3D image reconstruction are presented in the next section.

### Quantitative analysis

This study used the root-mean-square error (RMSE), peak signal-to-noise ratio (PSNR), and universal quality index (UQI) to evaluate the reconstruction results. These three quantitative indicators are defined as follows:

$$RMSE={\left\{\frac{1}{M\times N}\sum_{0\leq i<N}\sum_{0\leq j<M}{\left(f_{i,j}-{f_{i,j}}^{*}\right)}^{2}\right\}}^{\frac{1}{2}}$$ (13)

$$PSNR=20\;\log_{10}\left(\frac{MAX({\vec{f}}^{*})}{RMSE(\vec{f},{\vec{f}}^{*})}\right)$$ (14)

$$UQI=\frac{4\sigma_{\vec{f}{\vec{f}}^{*}}\bar{\vec{f}}\times \bar{{\vec{f}}^{*}}}{\left(\sigma_{\vec{f}}^{2}+\sigma_{{\vec{f}}^{*}}^{2})({\left(\bar{\vec{f}}\right)}^{2}+{\left(\bar{{\vec{f}}^{*}}\right)}^{2}\right)}$$ (15)

where *f*_*i*,*j*_ and *f*_*i*,j_* indicate the pixel values of the original and reconstructed images; *M* and *N* denote the total number of rows and columns in the images, respectively; $\sigma_{\vec{f}}^{2}$ and $\sigma_{{\vec{f}}^{*}}^{2}$ are the variance of $\vec{f}$ and ${\vec{f}}^{*}$; and $\sigma_{\vec{f}{\vec{f}}^{*}}$ is the covariance of $\vec{f}$ and ${\vec{f}}^{*}$.

### Experimental design

This study employed matlab2016 (Mathworks, Natick, MAUSA) to implement all the reconstruction algorithms. A 2D Shepp–Logan phantom and a 3D CT image of the human abdomen, which was downloaded from the open source "Cancer Imaging Archive" [46], were used as the ground truth to test the developed reconstruction method. The CT scan range was set at 360° in all reconstruction experiments. To ensure fairness of comparison, all parameters used were identical for these reconstruction algorithms: *Q* = 0.005; *Q*_*s*_ = 0.998; The parameter settings were based on Yu and Wang [32] and were modified according to our experiments.

There are three kinds of experiments. First, to observe the better performance of the proposed algorithms even when the iteration number is small, we set the iteration number for all iterative reconstruction algorithms to six, and compared the image quality among all the algorithms. Second, to examine the results approaching convergence in the 2D Shepp–Logan phantom experiment, we implemented all the algorithms until two thousand iterations, then compared the qualitative and quantitative results for all algorithms. The third experiment is to verify the applicability of applying the proposed multi-directional gradient operators to other CS-based algorithms. We choose the EPTV algorithm proposed by Tian Z et al [33] to combine with the proposed multi-directional gradient operators to reconstruct 2D Shepp-Logan phantom and 3D abdomen images for comparison.

In the 2D Shepp–Logan phantom reconstruction test, the results obtained using FBP, ART, and the 2-, 4-, and 8-directional gradient operators were compared for sampling intervals of 5° and 10°.

In the 3D human abdomen reconstruction test, projected images were captured at intervals of 5° and 10° to obtain two groups of sparse-view CT images (those images sampled with the same interval were categorized into the same group). Each group of images was reconstructed using FBP, ART, and the gradient operators with 3, 6, and 26 directions, and the results were compared. The 3-directional gradient operator calculates the gradients in the directions of three voxels, *f*_*i*−1,*m*,*n*_, *f*_*i*,*m*−1,*n*_, and *f*_*i*,*m*,*n*−1_ that are adjacent to *f*_*i*,*m*,*n*_. The 6-directional gradient operator calculates the gradients in the directions of six voxels, *f*_*i*+1,*m*,*n*_, *f*_*i*,*m*+1,*n*_, and *f*_*i*,*m*,*n*+1_, *f*_*i*−1,*m*,*n*_, *f*_*i*,*m*−1,*n*_, and *f*_*i*,*m*,*n*−1_ that are adjacent to *f*_*i*,*m*,*n*_.

## Results

### 2D Shepp–Logan phantom reconstruction results

**Fig 3:**
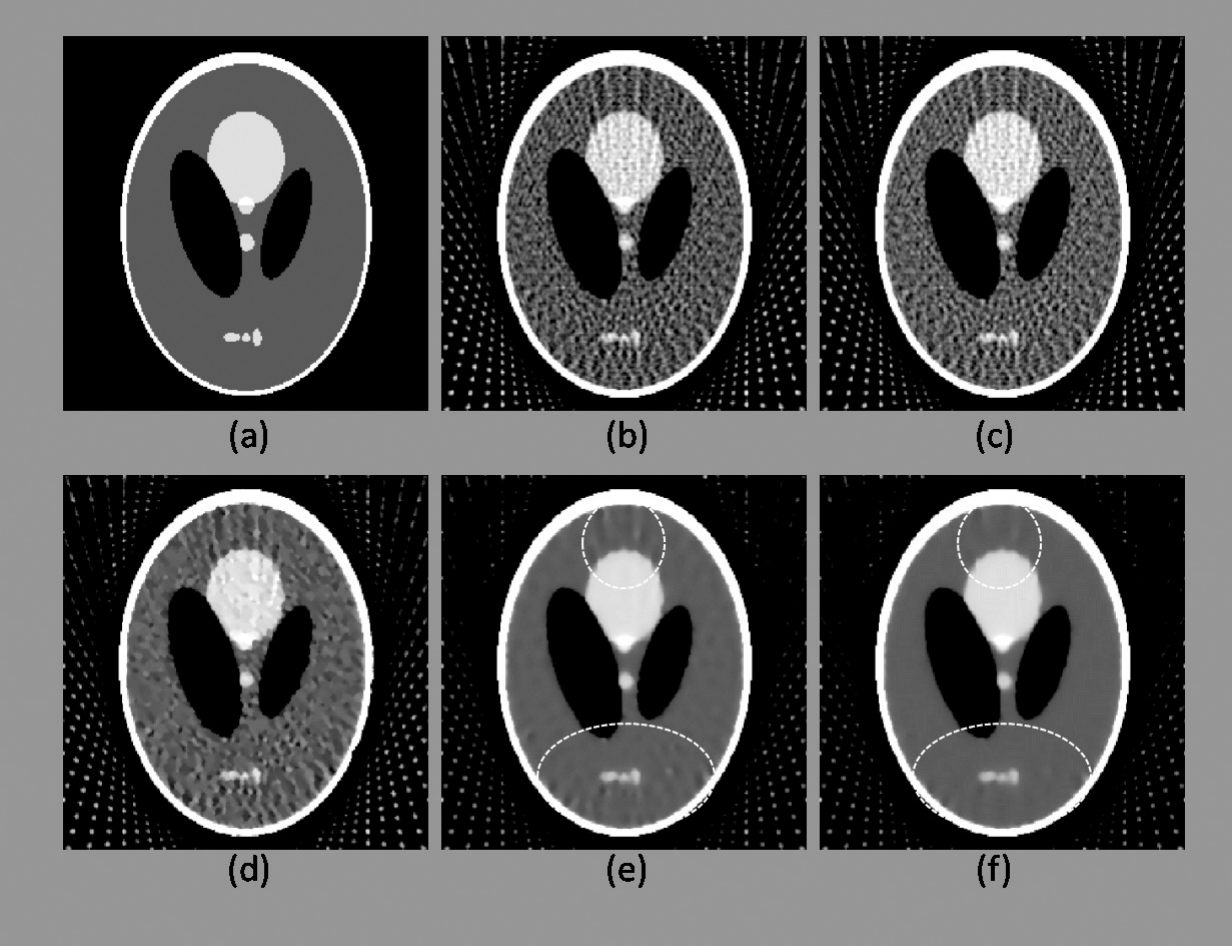
Reconstruction results after six iterations obtained for a sampling interval of 5°. (a) Original Shepp-Logan phantom. (b)–(f) Results reconstructed using FBP, ART, 2-, 4-, and 8-directional gradient operators, respectively. Areas marked by dotted ellipses are the differences of the results between 4-, and 8-directional gradient operators. As it can be seen in (f), when sampling interval is 5°, 8-directional gradient operator gave the fewest artifacts.

**Fig 4:**
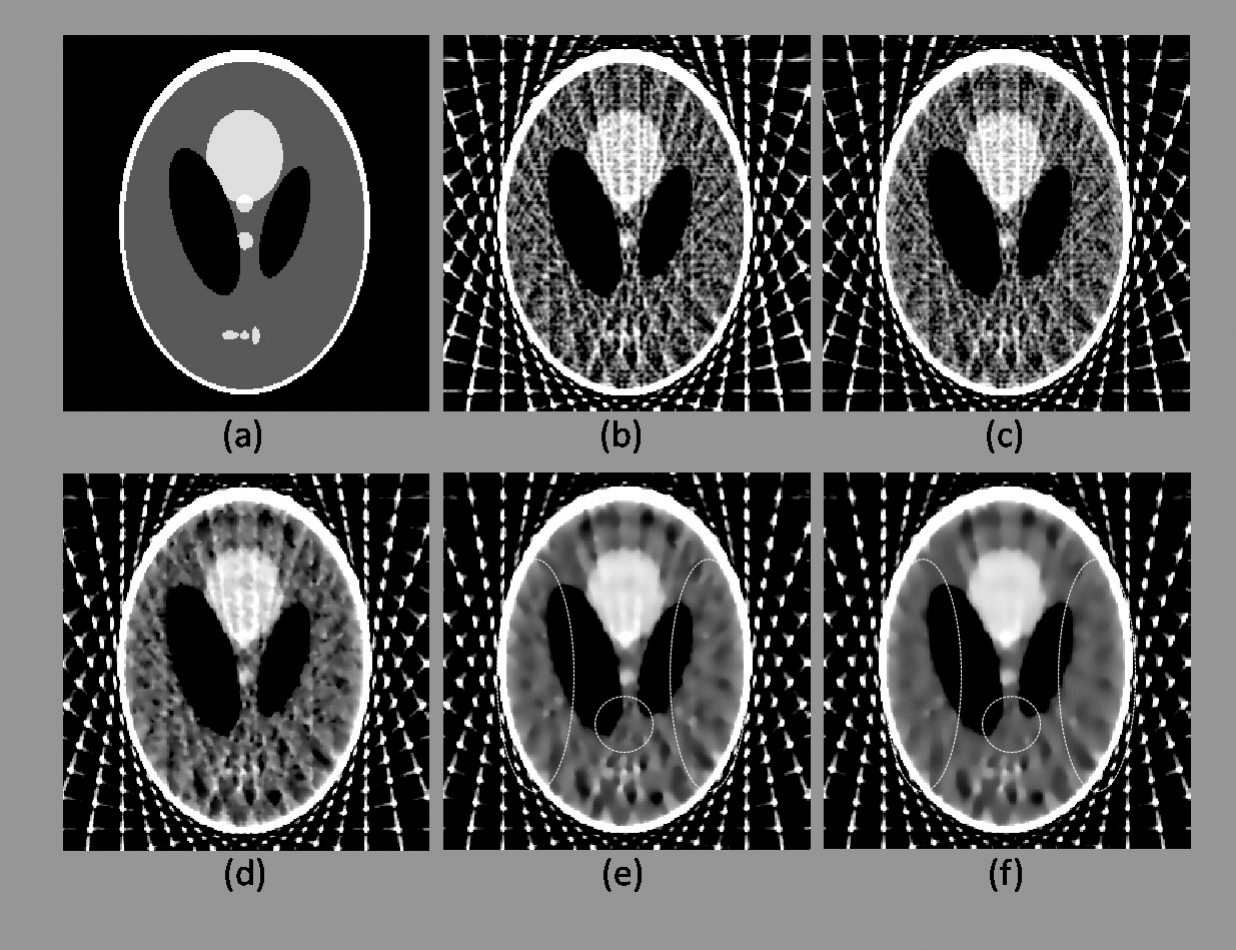
As in Fig 3, this figure shows the reconstruction results after six iterations obtained for a sampling interval of 10°. (a) Original Shepp-Logan phantom. (b)–(f) Results reconstructed using FBP, ART, 2-, 4-, and 8-directional gradient operators, respectively. Areas marked by dotted ellipses are the differences of the results between 4-, and 8-directional gradient operators. In this scanning circumstance, artifacts and noise in the reconstructed images are more vivid than Fig 3. However, the image obtained from 8-directional gradient operator still has the best image quality.

In the first experiment, the reconstruction results after six iterations are presented in Figs 3 and 4, and the quantitative analysis results are displayed in Tables 1 and 2.

**Table 1:** Quantitative analysis of the reconstructed Shepp–Logan phantom by using FBP, ART, 2-, 4-, and 8-directional gradient operators after six iterations for sampling interval of 5°. The image obtained from the 8-directional gradient operator has the best quantitative results.

**Method**	**Log(RMSE)**	**PSNR**	**UQI**
FBP	-0.989	19.79	0.882
ART	-0.999	19.99	0.887
2-directional gradient operator	-1.014.	20.28	0.893
4-directional gradient operator	-1.051	21.02	0.907
8-directional gradient operator	**-1.058**	**21.17**	**0.909**

Note: RMSE: root-mean-square error; PSNR: peak signal-to-noise ratio; UQI: universal quality index; FBP: filtered back projection; ART: algebraic reconstruction technique

**Table 2:** Quantitative analysis of the reconstructed Shepp–Logan phantom by using FBP, ART, 2-, 4-, and 8-directional gradient operators after six iterations for sampling interval of 10°.

**Method**	**Log(RMSE)**	**PSNR**	**UQI**
FBP	-0.749	14.98	0.713
ART	-0.806	16.13	0.755
2-directional gradient operator	-0.824	16.48	0.767
4-directional gradient operator	-0.848	16.97	0.785
8-directional gradient operator	**-0.856**	**17.12**	**0.789**

Note: As in Table 1, image obtained from the 8-directional gradient operator has the best quality. RMSE: root-mean-square error; PSNR: peak signal-to-noise ratio; UQI: universal quality index; FBP: filtered back projection; ART: algebraic reconstruction technique

According to Figs 3 and 4, the results obtained using the CS-based reconstruction algorithms were all superior to those obtained using the traditional FBP and ART. Results obtained using 2-, 4-, and 8-directional gradient operators were further compared. The image obtained using the 2-directional gradient operator has clear artifacts and noise when the iteration number was six, where as those obtained using the 4-directional gradient operator contain substantially fewer artifacts and less noise. The image reconstructed using the 8-directional gradient operator has the fewest artifacts and least noise. These findings can be observed in images (d)–(f) in Figs 3 and 4, especially in the areas marked by dotted ellipses. Quantitative analysis revealed the same results (Tables 1 and 2). Regardless of whether the sampling interval was 5° or 10°, the 2D Shepp–Logan phantom reconstructed using the 8-directional gradient operator exhibited the most satisfactory results in all three quantitative parameters compared with those reconstructed using other algorithms.

**Fig 5:**
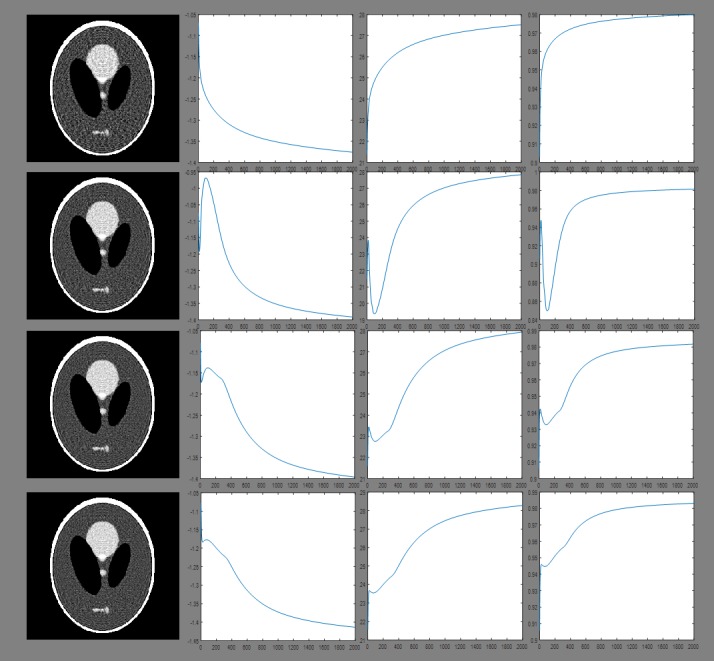
Reconstruction results approaching convergence obtained for a sampling interval of 5°. Columns from left to right show the reconstructed image, Log(RMSE), PSNR and UQI after two thousand iterations. Rows from top to bottom: images reconstructed using ART, 2-, 4-, and 8-directional gradient operators. When the algorithms use more number of directions in gradient operators, then all three figures of merit are better.

**Fig 6:**
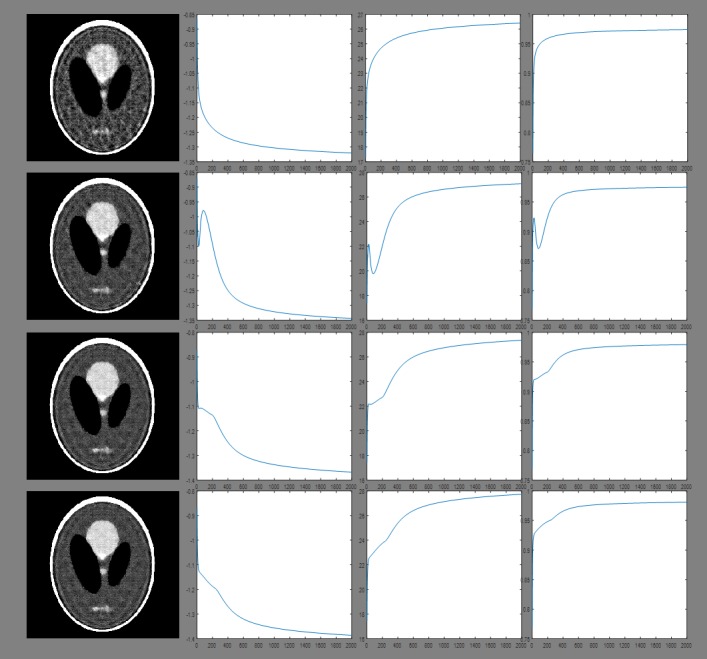
Reconstruction results approaching convergence obtained for a sampling interval of 10°. Columns from left to right show the reconstructed image, Log(RMSE), PSNR and UQI after two thousand iterations. Rows from top to bottom: images reconstructed using ART, 2-, 4-, and 8-directional gradient operators. As the same in Fig 5, even if the sampling interval is 10°, the gradient operators with more number of directions have better image quality.

The Second experiment is to compare the reconstructed images approaching convergence (after two thousand iterations). The results are presented in Figs 5 and 6, and the quantitative analysis results are displayed in Tables 3 and 4.

**Table 3:** Quantitative analysis of the reconstructed Shepp–Logan phantom by using ART, 2-, 4-, and 8-directional gradient operators after two thousands iterations for sampling interval of 5°.

**Method**	**Log(RMSE)**	**PSNR**	**UQI**
ART	-1.375	27.51	0.9800
2-directional gradient operator	-1.391	27.82	0.9813
4-directional gradient operator	-1.396	27.92	0.9817
8-directional gradient operator	**-1.413**	**28.27**	**0.9832**

Note: RMSE: root-mean-square error; PSNR: peak signal-to-noise ratio; UQI: universal quality index; FBP: filtered back projection; ART: algebraic reconstruction technique

**Table 4:** Quantitative analysis of the reconstructed Shepp–Logan phantom by using ART, 2-, 4-, and 8-directional gradient operators after two thousands iterations for sampling interval of 10°.

**Method**	**Log(RMSE)**	**PSNR**	**UQI**
ART	-1.320	26.41	0.9740
2-directional gradient operator	-1.367	27.35	0.9791
4-directional gradient operator	-1.373	27.47	0.9798
8-directional gradient operator	**-1.386**	**27.72**	**0.9808**

Note: RMSE: root-mean-square error; PSNR: peak signal-to-noise ratio; UQI: universal quality index; FBP: filtered back projection; ART: algebraic reconstruction technique

From the second experiment, the results from all the algorithms approaching convergence can be found after two thousand iterations. Above results are illustrated in Figs 5 and 6 and Tables 3 and 4. The RMSE obtained from 8-directional gradient operator has the lowest value, and the corresponding PSNR and UQI have the highest value. Similarly, 4-directional gradient operator has better results than 2-directional gradient operator. The worst one is the results of ART algorithm.

In order to verify the applicability of applying the proposed multi-directional gradient operators to other CS-based algorithms, we perform the third experiment which combines the previous EPTV algorithm [33] with the proposed multi-directional gradient operators to reconstruct the Shepp-Logan phantom. The results are as follows.

**Fig 7:**
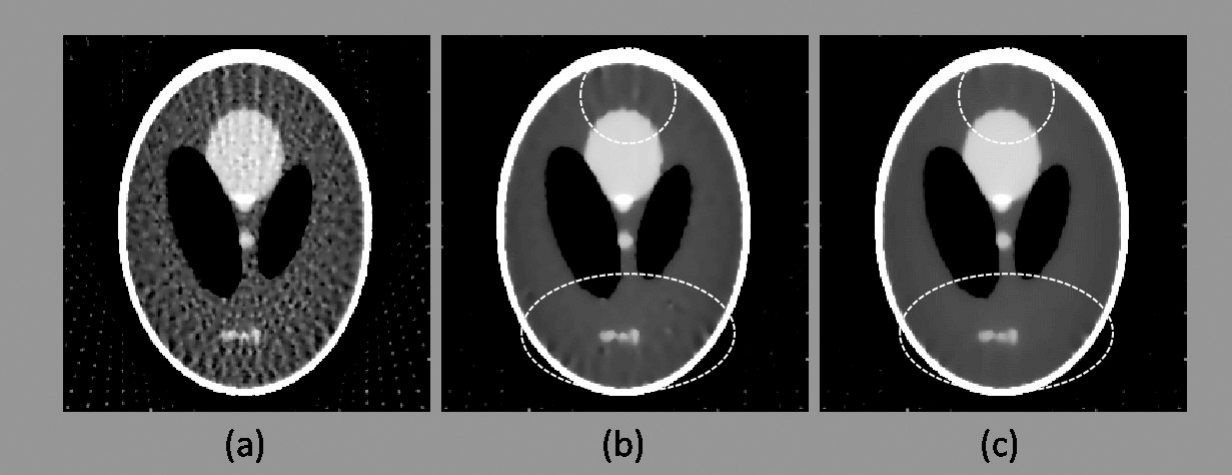
Reconstruction results of Shepp-Logan phantom by using EPTV combining with the multi-directional gradient operators when the sampling interval is 5°. (a)–(c) Results reconstructed using EPTV combining with 2-, 4-, and 8-directional gradient operators, respectively. Areas marked by dotted ellipses are the differences of the results between 4-, and 8-directional gradient operators. Even if combined with EPTV, the images reconstructed from more number of directions in gradient operators still have less artifacts.

As shown in Fig 7 and Table 5, these results are consistent with our first and second experiments: the more number of directions in gradient operators, the better images will be reconstructed, even if our proposed algorithm is combined with other CS-based method.

**Table 5:** Quantitative analysis of the reconstructed Shepp–Logan phantom by using EPTV combined with 2-, 4-, and 8-directional gradient operators, when the sampling interval is 5°.

**Method**	**Log(RMSE)**	**PSNR**	**UQI**
EPTV + 2-directional gradient operator	-1.179	23.09	0.9389
EPTV + 4-directional gradient operator	-1.195	23.90	0.9491
EPTV + 8-directional gradient operator	-1.208	24.36	0.9546

### 3D CT image reconstruction results

**Fig 8:**
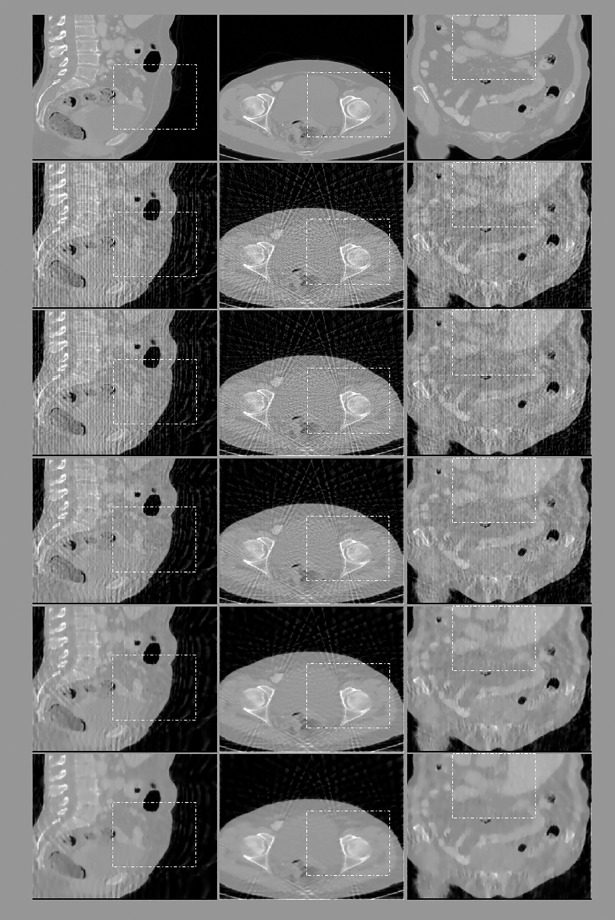
Reconstruction results of an abdomen image after six iterations obtained for a sampling interval of 5°. First row: ground truth; subsequent rows from top to bottom: images reconstructed using FDK, ART, and the 3-, 6-, and 26-directional gradient operators. Images from left to right show the sagittal, transaxial, and coronal sections of the abdomen. Areas marked by dotted rectangles are enlarged and displayed in Fig 9. Subsequent rows from top to bottom, the lower images in the figure, the smoother they are, and are closer to the original images.

**Fig 9:**
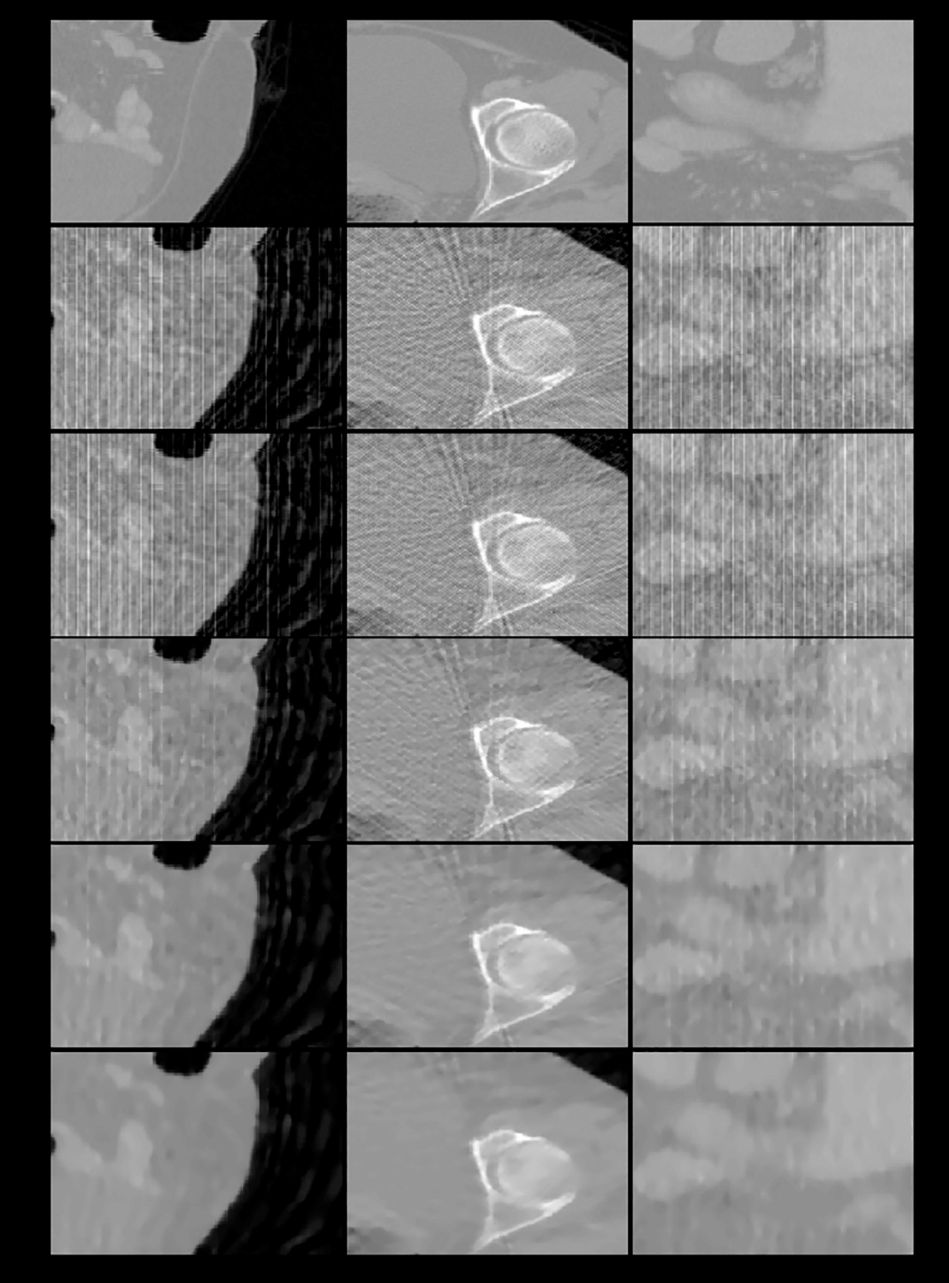
The zoom-in views of the images displayed in previous one figure. First row: ground truth; subsequent rows from top to bottom: images reconstructed using FDK, ART, and the 3-, 6-, and 26-directional gradient operators. Subsequent rows from top to bottom, the more number of directions in gradient operators, the less streak artifacts the reconstructed images have.

**Fig 10:**
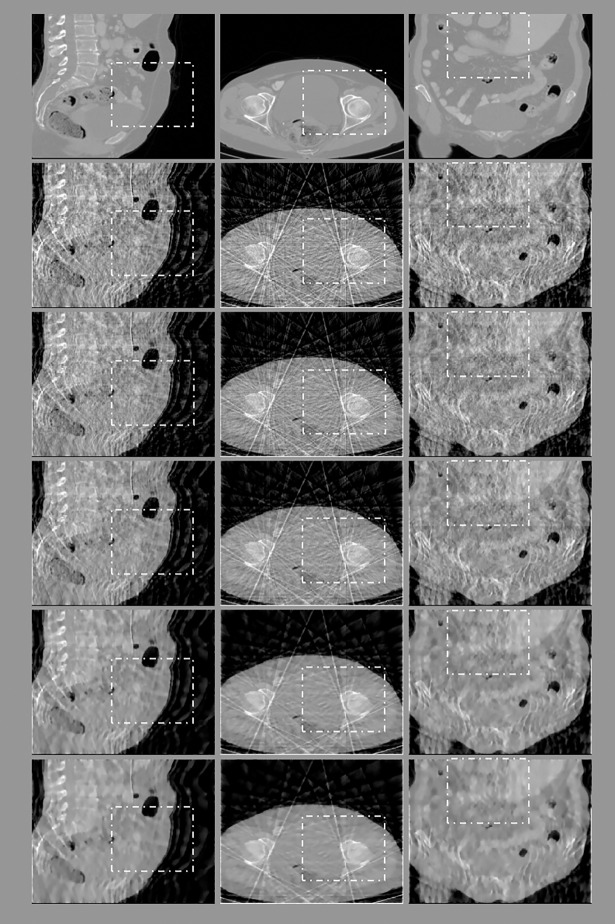
Reconstruction results of an abdomen image after six iterations obtained for a sampling interval of 10°. First row: ground truth; subsequent rows from top to bottom: images reconstructed using FDK, ART, and the 3-, 6-, and 26-directional gradient operators. Images from left to right show the sagittal, transaxial, and coronal sections of the abdomen. Areas marked by dotted rectangles are enlarged and displayed in Fig 11. Artifacts in the reconstructed images are more obvious than Fig 8. However, as seen in Fig 8, subsequent rows from top to bottom, the lower images in the figure, the smoother they are.

**Fig 11:**
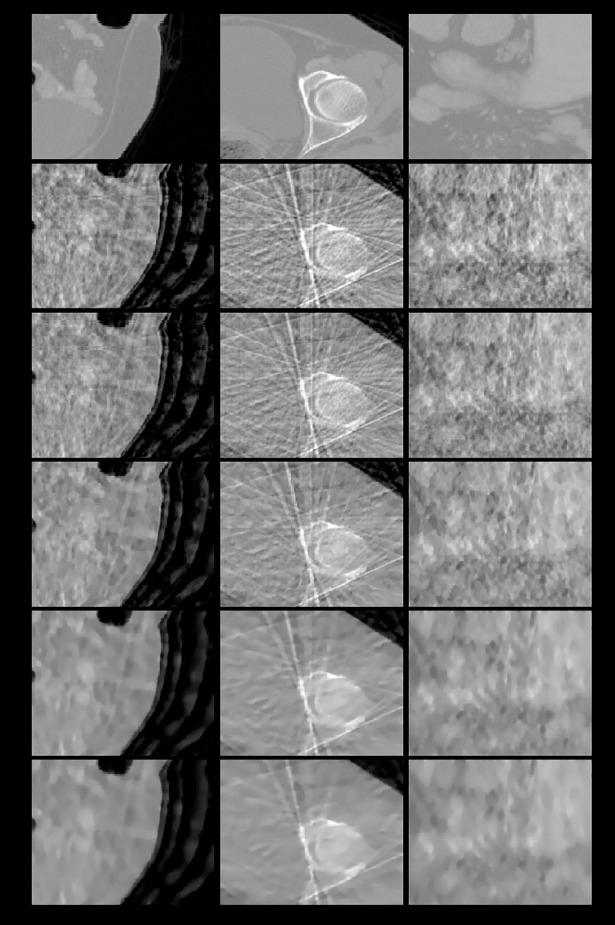
The zoom-in views of the images displayed in Fig 10. As the same in Figs 8–10, the images reconstructed from the 26-directional gradient operators have the least artifacts and noise.

In the experiment to observe the better performance of the proposed algorithms even when the iteration number is small, the reconstruction results after six iterations are presented in Figs 8–11, and the quantitative analysis results are displayed in Tables 6 and 7.

**Table 6:** Quantitative analysis of the abdomen image reconstructed using FBP, ART, 3-, 6-, and 26-directional gradient operators after six iterations for sampling interval of 5°.

**Method**	**RMSE**	**PSNR**	**UQI**
Sagittal section			
FBP	140.46	24.21	0.9589
ART	131.51	24.78	0.9637
3-directional gradient operator	97.89	27.34	0.9795
6-directional gradient operator	78.21	29.29	0.9868
26-directional gradient operator	**72.29**	**29.98**	**0.9886**
Transaxial section			
FBP	147.85	24.13	0.9583
ART	135.58	24.88	0.9647
3-directional gradient operator	116.74	26.18	0.9735
6-directional gradient operator	100.56	27.48	0.9802
26-directional gradient operator	**95.86**	**27.89**	**0.9819**
Coronal section			
FBP	151.43	23.49	0.9028
ART	140.51	24.14	0.9154
3-directional gradient operator	111.37	26.16	0.9447
6-directional gradient operator	91.85	27.83	0.9615
26-directional gradient operator	**86.28**	**28.38**	**0.9656**

Note: The images obtained from 26-directional gradient operator have the best quantitative results. RMSE: root-mean-square error; PSNR: peak signal-to-noise ratio; UQI: universal quality index; FBP: filtered back projection; ART: algebraic reconstruction technique

**Table 7:** Quantitative analysis of the abdomen image reconstructed using FBP, ART, 3-, 6-, and 26-directional gradient operators after six iterations for sampling interval of 10°.

**Method**	**RMSE**	**PSNR**	**UQI**
Sagittal section			
FBP	204.37	20.95	0.9183
ART	173.91	22.35	0.9390
3-directional gradient operator	160.75	23.03	0.9473
6-directional gradient operator	140.06	24.23	0.9592
26-directional gradient operator	**131.01**	**24.81**	**0.9640**
Transaxial section			
FBP	260.42	19.21	0.8790
ART	214.12	20.91	0.9151
3-directional gradient operator	203.28	21.36	0.9225
6-directional gradient operator	187.04	22.09	0.9335
26-directional gradient operator	**180.47**	**22.40**	**0.9377**
Coronal section			
FBP	232.08	19.78	0.7930
ART	193.75	21.35	0.8460
3-directional gradient operator	183.42	21.83	0.8581
6-directional gradient operator	162.40	22.88	0.8849
26-directional gradient operator	**154.44**	**23.32**	**0.8940**

Note: As in Table 3, the image quality of the images obtained from 26-directional gradient operator is the best. RMSE: root-mean-square error; PSNR: peak signal-to-noise ratio; UQI: universal quality index; FBP: filtered back projection; ART: algebraic reconstruction technique

Two observations can be obtained from the results of the 3D abdomen image reconstruction. First, the results obtained using the CS-based reconstruction algorithms were all superior to those obtained using the FDK and ART algorithms, regardless of the number of directions used for TV calculation. Second, the more number of directions the gradient operator used for TV calculation, the better quality the reconstructed images, as exhibited by both qualitative and quantitative analyses. These two observations are valid regardless of whether the sampling interval was 5° or 10° and whether the images were sagittal, transaxial, or coronal sections.

**Fig 12:**
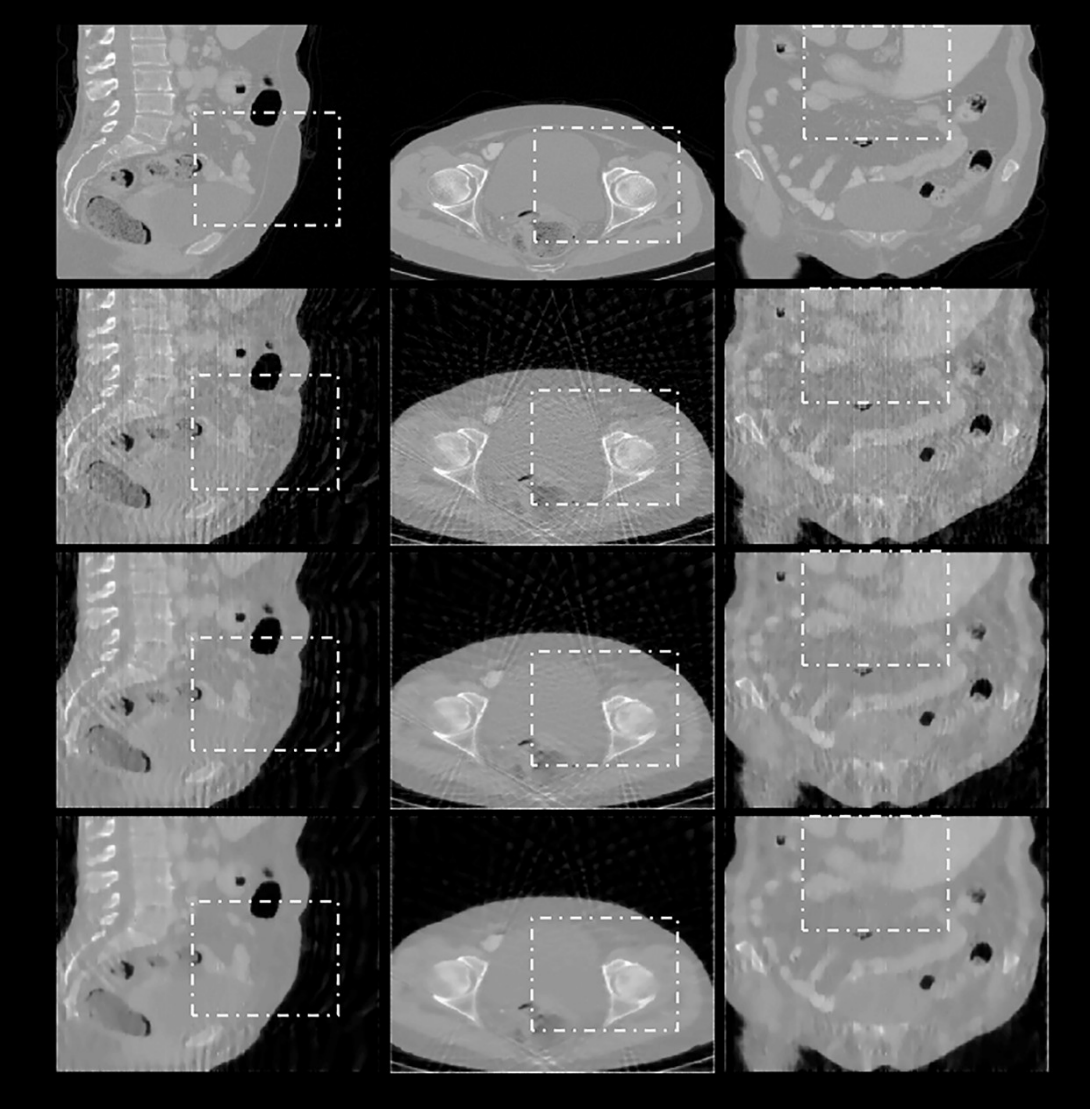
Reconstruction results of an abdomen image by using EPTV combined with the multi-directional gradient operators when the sampling interval is 5°. First row: ground truth; subsequent rows from top to bottom: images reconstructed using EPTV combined with the 3-, 6-, and 26-directional gradient operators, respectively. Images from left to right show the sagittal, transaxial, and coronal sections of the abdomen. Areas marked by dotted rectangles are enlarged and displayed in Fig 13. Subsequent rows from top to bottom, the lower images in the figure, the smoother they are, and are closer to the original images.

**Fig 13:**
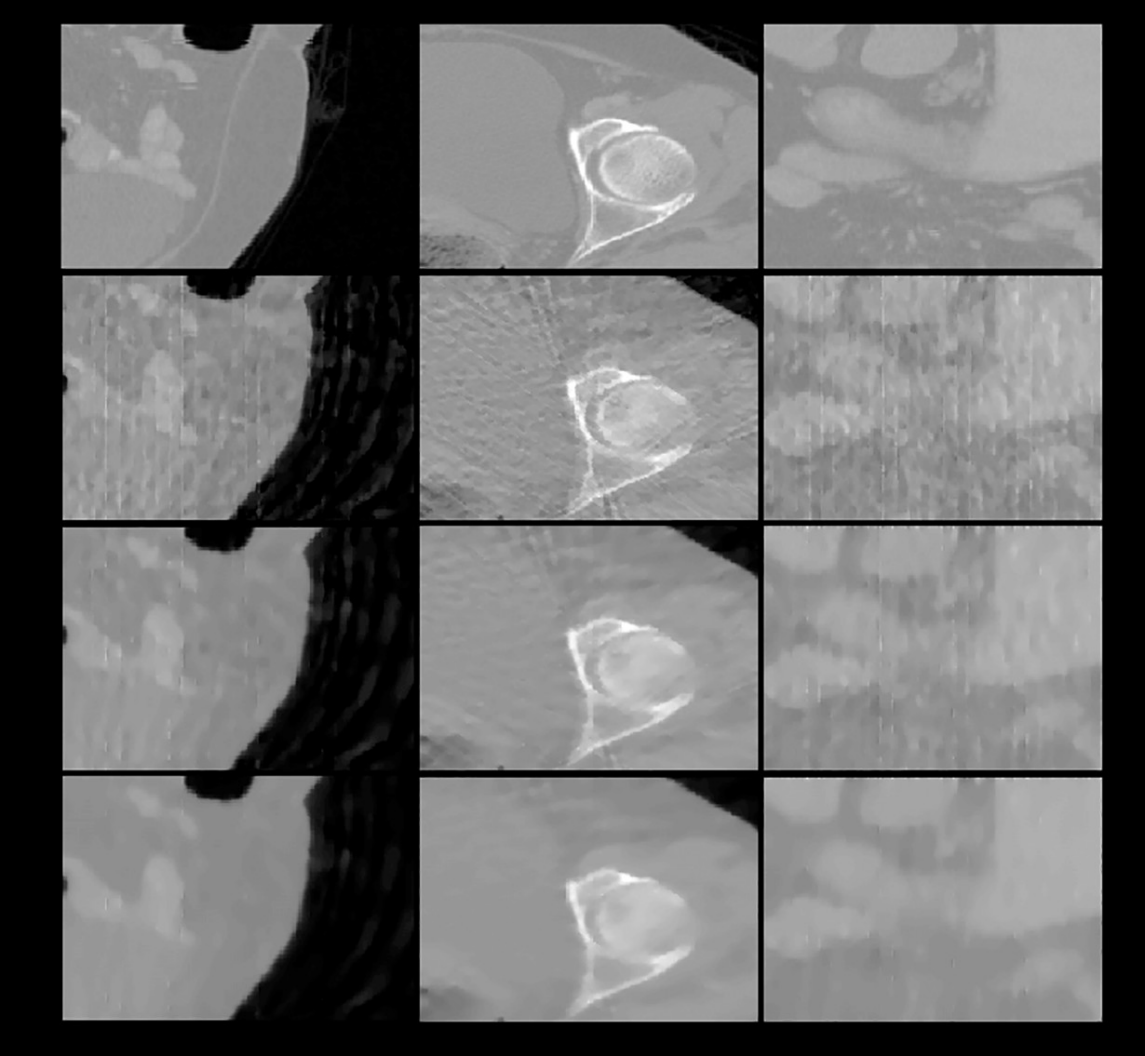
The zoom-in views of the images displayed in Fig 12. First row: ground truth; subsequent rows from top to bottom: images reconstructed using EPTV combined with the 3-, 6-, and 26-directional gradient operators. Even if combined with EPTV, the more number of directions in gradient operators, the less streak artifacts the reconstructed images have.

To confirm the applicability of combining the proposed multi-directional gradient operators with existing CS-based algorithms, we also use the 3D abdomen image to test the algorithm which is combined with EPTV method. The results are presented in Figs 12 and 13.

As shown in Figs 12 and 13 and Table 8, even in the 3D reconstruction circumstance, EPTV combined with 26-directional gradient operator has the best image quality. The second one is the results of combining with 6-directional gradient operator, and so on. Therefore, the proposed multi-directional gradient operators can be applied to and combined with the EPTV algorithm.

**Table 8:** Quantitative analysis of the reconstructed abdomen images by using EPTV combined with 3-, 6-, and 26-directional gradient operators, when the sampling interval is 5°.

**Method**	**RMSE**	**PSNR**	**UQI**
Sagittal section			
3-directional gradient operator	91.84	27.90	0.9818
6-directional gradient operator	74.12	29.76	0.9881
26-directional gradient operator	**69.16**	**30.36**	**09895**
Transaxial section			
3-directional gradient operator	109.27	26.76	0.9767
6-directional gradient operator	94.29	28.04	0.9825
26-directional gradient operator	**90.40**	**28.40**	**0.9839**
Coronal section			
3-directional gradient operator	104.64	26.70	0.9506
6-directional gradient operator	86.89	28.32	0.9653
26-directional gradient operator	**81.94**	**28.83**	**0.9688**

## Discussion

Sidky and Pan proved that minimizing the L1-norm of TV in iterative reconstruction process could effectively remove high frequency parts such as noise and artifacts caused by few-view situation in CT imaging. But in their studies, TV method only calculated the total variation (TV) in x and y directions to obtain a sparse representation in reconstruction process. We reason that if we consider the calculation of TV in both x, y directions and the diagonal directions for the gradient transform, it will preserve more information from original tomographic data in the iteration process and make the reconstructed results closer to the original image. To verify this idea, we have proposed 8-directional gradient operator and 26-directional gradient operator for 2D and 3D reconstruction, respectively. And then compare the results obtained from other algorithms to emphasize the better performance of reducing noise and artifacts by applying our algorithm.

We have performed three kinds of experiments to confirm our reasoning. First experiment is to assure the better performance even when the iteration number is small. As shown in Figs 3 and 4 and Figs 8–11, whether the targets are 2D Shepp-Logan phantom or 3D abdomen image and the sampling interval is 5° or 10°, the results from 8-directional and 26-directional gradient operator have the least artifacts and are closest to original image when the iteration number is six. The second best algorithm in this experiment is 4-directional and 6-directional gradient operator, the reconstructed images from them are visually clearer than 2-directional and 3-directional gradient operator. Quantitative analysis also reflects the same results in Tables 1 and 2 and Tables 6–7.

To compare the results approaching convergence, we used the Shepp-Logan phantom as the target and performed two thousands iterations in all the reconstruction algorithms in the second experiment. The results can be seen in Figs 5 and 6 and Tables 3 and 4. According to the curve of RMSE, PSNR and UQI in Figs 5 and 6, the results of all the algorithms approaching convergence can be found when the iteration number reaches two thousand. Tables 3 and 4 show the quantitative results of all algorithms after two thousand iterations. Both of the qualitative and quantitative analyses show that 8-directional gradient operator has the best results, followed by 4-directional gradient operator. This conclusion is the same as the first experiment.

The final goal of our study is to apply the 8-directional and 26-directional gradient operators to other CS-based algorithms to further improve the quality of reconstructed images. To fulfill above goal, the third experiment combined the proposed multi-directional gradient operators with EPTV to verify the applicability. We first compare the reconstructed images in Figs 3 and 7. At the same image testing conditions, the results from the proposed multi-directional gradient operators combined with EPTV are visually clearer and have better contrast in edges than the results only used the traditional EPTV. The quantitative analysis in Tables 1 and 5 also reflect the same results. Next, from Fig 7 and Table 5, we also notice that more number of directions in gradient operators combined with EPTV have better results. Similarly, the image quality of the results from EPTV combined with 8-directional gradient operator is the best. In 3D abdomen image reconstruction test, the results are the same as 2D Shepp–Logan phantom test. From the results of the third experiment, we have verified that 8-directional and 26-directional gradient operators can be effectively applied to EPTV algorithm.

These three experiments verify that increasing the number of directions in TV calculation actually improve the image quality of reconstruction. Additional consideration of the TV calculation in diagonal direction, like 8-directional and 26-directional gradient operator, effectively preserve more information from original tomographic data in the reconstruction process and make the reconstructed results better than those of previous TV method. Another advantage of the proposed method is that it is applicable to be applied to combine with other algorithms derived from CS theory. In this study, we have successfully applied the 8-directional and 26-directional gradient operator to the EPTV algorithm. In the future, we will try to apply the proposed method to other CS-based algorithm, like PICCS (prior image constrained compressed sensing)[31], AwTV (adaptive-weighted TV)[34], NPICCS (Nonconvex prior image constrained compressed sensing)[47] etc., to further confirm the applicability of our proposed method.

The effectiveness and applicability of the proposed multi-directional gradient operators in sparse-view CT reconstruction have been proven. However, the calculation of our proposed method are much more complex than other reconstruction methods discussed in this study. Therefore, multi-directional gradient operators are the most time-consuming compared with all other reconstruction algorithms (the computational costs are compared in Tables 9 and 10). To deal with this problem, we expect to modify the proposed algorithm into graphics processing unit (GPU) format to accelerate the reconstruction process in the future.

**Table 9:** The reconstruction time of each iteration in the experiment of 2D Shepp-Logan phantom.

	**2-directional TV**	**4-directional TV**	**8-directional TV**
Time	0.11s	0.14s	0.15s

**Table 10:** The reconstruction time of each iteration in the experiment of 3D abdomen image.

	**3-directional TV**	**6-directional TV**	**26-directional TV**
Time	121.5s	176.0s	2084.2s

## Conclusions

This paper proposes the use of multi-directional gradient operators to improve the quality of CT images reconstructed using CS-based algorithms. A 2D Shepp–Logan phantom and 3D clinical abdomen images were employed to test and verify that the 2D and 3D images reconstructed using 8- and 26-directional gradient operators, respectively, have higher image quality than those reconstructed using the traditional 2- or 4- and 3- or 6-directional gradient operators. The proposed multi-directional gradient operator algorithms have high potential to be applied to and combined with existing CT reconstruction algorithms derived from CS theory to produce better image quality in sparse-view reconstruction.

## Supporting information

S1 File 2D Shepp–Logan phantom. Original Shepp–Logan phantom image for our 2D reconstruction test.
(RAR)

S2 File 3D clinical abdomen images. Original clinical abdomen images downloaded from The Cancer Imaging Archive http://www.cancerimagingarchive.net/ for our 3D reconstruction test.
(RAR)
